# No *PfATPase6 *S769N mutation found in *Plasmodium falciparum *isolates from China

**DOI:** 10.1186/1475-2875-7-122

**Published:** 2008-07-08

**Authors:** Guoqing Zhang, Yayi Guan, Bin Zheng, Song Wu, Linhua Tang

**Affiliations:** 1 National Institute of Parasitic Diseases, Chinese Center for Disease Control and Prevention, 207 Rui Jin Er Road, Shanghai, 200025, PR China

## Abstract

**Background:**

Artemisinin and its derivatives have been used for falciparum malaria treatment in China since late 1970s. Monotherapy and uncontrolled use of artemisinin drugs were common practices for a long period of time. *In vitro *tests showed that the susceptibility of *Plasmodium falciparum *to artemisinins was declining in China. A concern was raised about the resistance to artemisinins of falciparum malaria in the country. It has been reported that *in vitro *artemisinin resistance was associated with the S769N mutation in the *PfATPase6 *gene. The main purpose of this study was to investigate whether that mutation has occurred in field isolates from China.

**Methods:**

*Plasmodium falciparum *field isolates were collected in 2006–2007 from Hainan and Yunnan provinces, China. A nested PCR-sequencing assay was developed to analyse the genotype of the *PfATPase6 *S769N polymorphism in the *P. falciparum *field isolates.

**Results:**

The genotyping results of six samples could not be obtained due to failure of PCR amplification, but no S769N mutation was detected in any of the 95 samples successfully analysed.

**Conclusion:**

The results indicate that the S769N mutation in the *PfATPase6 *gene is not present in China, suggesting that artemisinin resistance has not yet developed, but the situation needs to be watched very attentively.

## Background

China has been severely afflicted by falciparum malaria. In 1954, falciparum malaria cases totaled about 4.18 million in the whole country. With active implementation of malaria control measures for more than 50 years, considerable success has been achieved [1]. However, up to the present, falciparum malaria is still an important public health problem in China, especially in Yunnan and Hainan provinces, with 3,240 cases reported from the two provinces in 2006, accounting for 93.4% of the total reported cases in the country [2].

The plant *Artemisia annua *(Asteraceae) has been used for more than 2,000 years in Chinese traditional medicine for the treatment of febrile illnesses, including malaria. *Artemisia annua *contains artemisinin, which was isolated in 1972, and since that time its efficacy against malaria has been amply demonstrated [3,4]. Following increased chloroquine (CQ), piperaquine (PQ) and pyrimethamine-sulphadoxine (SP) resistance, artemisinin and its derivatives gradually became the mainstay of falciparum malaria therapy in China. Before the World Health Organization (WHO) urged pharmaceutical companies to end the marketing and sale of artemisinin for monotherapy in 2006 [5], many drug manufacturers in China distributed artemisinin alone to treat malaria; indiscriminate use of artemisinin derivatives was common practice. Therefore, whether resistance to artemisinins has developed in China is a cause for concern.

To this day, clinically relevant artemisinin resistance has not yet been documented, but it was found that the susceptibility of *Plasmodium falciparum *to artemisinin derivatives was declining in China, as IC50 for artesunate in 1999 were 3.3 times of that in 1988 [6]. Laboratory studies have shown that genetically stable and transmissible artemisinin-resistant rodent malaria parasite could be selected through prolonged exposure of drug-sensitive lines to low and increasing levels of artemisinins [7]. It is likely a matter of time before clinical artemisinin resistance is observed in China.

Recent studies provide compelling evidences that artemisinins act by selectively inhibiting PfATPase6 protein, the only SERCA-type Ca^2+^-ATPase in the *P. falciparum *genome, believed to be the primary target for artemisinins [8,9]. A subsequent study in French Guyana showed that S769N *PfATPase6 *mutation was associated with raised artemether IC50, and suggested that the mutation may be used in molecular monitoring of artemisinin resistance to complement continuing *in-vitro *surveillance [10]. So far, no studies on this molecular marker have been carried out in China. The main purpose of the present study was to investigate whether there are any changes at codon S769N of the *PfATPase6 *locus in field samples from the country following the long-term use of artemisinins. A nested PCR-sequencing assay was developed to analyse the mutation of *PfATPase6 *S769N among *P. falciparum *field isolates collected from Hainan and Yunnan provinces, China.

## Methods

### Samples

**Figure 1:**
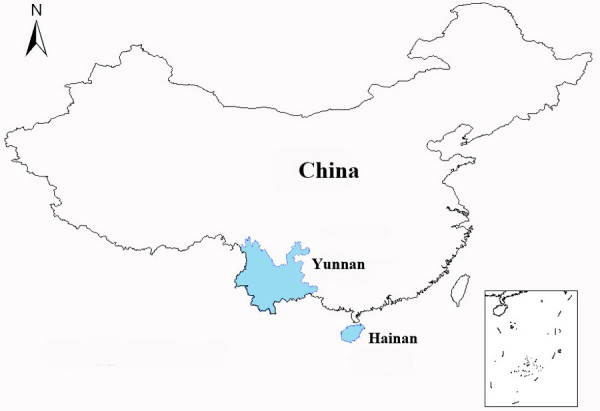
Map of China showing the locations (shadow) where parasite samples were collected.

*Plasmodium falciparum *field isolates were collected in 2006–2007 from two provinces. 27 samples were collected from Hainan province and 74 samples were from Yunnan province (Figure 1). The two provinces contributed more than 90% of the total falciparum malaria cases in the whole country in 2006 [2]. Parasite samples were collected from patients with uncomplicated *P. falciparum *infections before drug treatment. Diagnosis was carried out by microscopic examination of Giemsa-stained thick blood films. For each sample, approximately 20 μL of finger-prick blood was spotted on a piece of 3 MM filter paper (Whatman, Maidstone, UK) and air-dried. The dried filter paper samples were stored in individual zipper plastic bags with dryer at -20°C until DNA extraction. This study was reviewed and approved by the ethics committee of the National Institute of Parasitic Diseases, Chinese Center for Disease Control and Prevention.

### DNA extraction

Extraction of DNA from bloodspots on filter paper was carried out by the Chelex-100 (Bio-Rad Laboratories, Hercules, CA) boiling method described by Wooden and others [11] with some modifications described by Pearce and others [12]. The quality of DNA samples was tested by OD 260/280 measurement.

### Nested PCR-sequencing assay

The fragment of *pfATPase6 *was amplified by nested PCR with primer F1 (5'-CAAATAAGAAGGATAAATCACCA-3') and primer R1(5'-TCATAAATACACGTATACCAGCC-3') (94°C for 3 min; then 94°C for 40 s, 46°C for 1 min, and 72°C for 1 min for 35 cycles; followed by a final extension step at 72°C for 10 min), and then primer F2 (5'-AAAATAAATACCACATCAACACAT-3') and primer R2 (5'-TCAATAATACCTAATCCACCTAAA-3') (94°C for 3 min; then 94°C for 40 s, 45°C for 1 min, and 72°C for 1 min for 35 cycles; followed by a final extension step at 72°C for 10 min). The 50 μL PCR mix contained primers at 0.25 μmol/L final concentration, 2 mmol/L MgCl_2_, 240 μmol/L of each deoxynucleoside triphosphate, and 2 U *Taq *polymerase. Template DNA (1 μL) was introduced to outer reaction mixtures. 1 μL outer PCR product was introduced into a 50 μL inner amplification mixture. Secondary PCR products were resolved by electrophoresis on 1% agarose gels and visualized by staining with ethidium bromide. Sequencing reactions were carried out using ABI PRISM Big Dye terminator v3.1 Cycle Sequencing kit (Applied Biosystems, CA, USA) as specified by the manufacturer's protocol. The sequences of the amplicons were aligned with the published data of the NCBI database by BLAST analysis.

## Results

### Nested PCR

**Figure 2:**
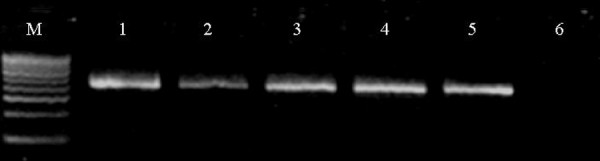
**Agarose gel electrophoresis of secondary PCR products**. M, 100 bp DNA ladder. Lanes 1–5, *P. falciparum *samples; lane 6: Blank control.

A 437-base pair fragment in the *PfATPase6 *gene was amplified by nested PCR in field samples. Gel electrophoresis of the secondary PCR products indicated that they were of the expected sizes (Figure 2). Under this amplification conditions, 6 out of 101 samples could not be amplified.

### Sequencing assay

**Figure 3:**
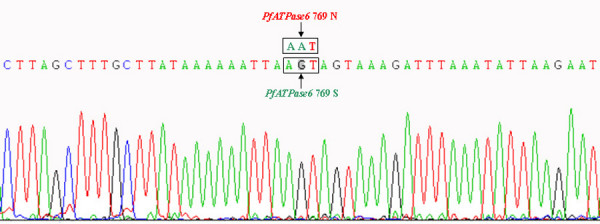
Sequencing image of nested PCR product indicating the polymorphism at codon 769 of the *PfATPase6 *gene.

Sequencing assays were successfully performed for the nested PCR products of 95 samples, no S769N mutation was detected in any of the analysed samples. Figure 3 shows a sequencing image of nested PCR product, indicating the polymorphism at codon 769 of the *PfATPase6 *gene.

## Discussion

In this study, by using nested PCR-sequencing assay, no *PfATPase6 *S769N mutation was found in any *P. falciparum *isolates collected from China. The finding is consistent with the results from the *in vitro *microtests [13,14] and the high (more than 96%) cure rates of artemisinin combination therapies (ACTs) treatment for falciparum malaria in China [15,16]. Accordingly, until now there is no sufficient evidence for assuming that artemisinin resistance has occurred in China. However, stable resistance to artemisinins has been induced in the laboratory, and the declining susceptibility of *P. falciparum *to artemisinin derivatives has been reported in the country [6]. Artemisinin drugs are believed as the most effective and the last hope in the near future in the battle against multidrug-resistant malaria, if resistance to artemisinins develops and spreads, it will be the most devastating event in the history of malaria control [17]. Therefore, it is wise to phase out artemisinin monotherapy in China; the use of ACTs recommended by WHO as first line treatment for uncomplicated malaria will either prevent, or at least delay, the development of artemisinin resistance.

Although evidences from laboratory and field studies have suggested that PfATPase6 protein is the primary target for artemisinins, and a specific S769N mutation in the gene was significantly associated with the reduced efficacy of artemisinins [8-10]. It should be pointed out that one laboratory study could not identify such mutation in artemisinin-resistant *Plasmodium chabaudi *[7]. Field studies carried out in Tanzania and Brazil also could not detect the S769N *PfATPase6 *mutation in the analysed samples [18,19]. Thus, whether this mutation can be widely used as a reliable marker for artemisinin resistance in epidemiological studies needs further validation.

## Conclusion

*PfATPase6 *S769N mutation was not detected in any of the *P. falciparum *isolates from China, no direct evidence supports that artemisinin resistance has occurred in the country until now. However, a high degree of vigilance is required; the level of artemisinin sensitivity of *P. falciparum *should be closely monitored.

## Authors' contributions

GZ performed laboratory work and wrote the manuscript. YG and BZ performed the field work. SW performed laboratory work. LT was involved in all stages of this study.
